# Supplementary material for: Attitude sustains longer than subjective norm and perceived behavioral control: Results of breast cancer screening educational intervention
Source: PLoS One. 2023 Feb 10;18(2):e0281184. doi: 10.1371/journal.pone.0281184 (PMC9916560; doi:10.1371/journal.pone.0281184)
Supplement: Attachment — Submitted filename: PONE-D-22-05192.pdf [file pone.0281184.s002.pdf]

PLOS ONE

The effect of educational intervention on intention to perform breast self-examination and mammography among women of Pokhara Nepal: application of theory of planned behavior

--Manuscript Draft--

**Manuscript Number:**	PONE-D-22-05192
**Article Type:**	Research Article
**Full Title:**	The effect of educational intervention on intention to perform breast self-examination and mammography among women of Pokhara Nepal: application of theory of planned behavior
**Short Title:**	Intention to perform breast self examination and mammography among women of Pokhara Nepal
**Corresponding Author:**	Rojana Dhakal<br>Pokhara University<br>Pokhara, Gandaki NEPAL
**Keywords:**	Breast self-examination; Intention; Mammography; Nepal; Theory of planned behavior
**Abstract:**	Breast malignancy is the most frequent carcinoma among females across the world. Early diagnosis of breast cancer by breast health awareness and self-examination with mammography screening is a highly feasible and useful technique in poor resource countries. The primary objective of this study was to assess how an educational intervention affected women’s intention to do breast self-examination and mammography screening in selected wards in Pokhara metrocity of Nepal. A non-randomized intervention was employed among 360 women (180 in intervention and 180 in control group) of 40-75 years. First, wards were randomly assigned in the intervention and control groups, then participants who satisfied inclusion criteria were purposefully chosen. Study participants in the intervention group were given a one-hour breast cancer screening related session. Also, respondents in the comparison group were given the similar educational session at the completion of the study. Relevant data was obtained at the baseline and, 4, 8 and 12 months of intervention. The intention to undertake breast carcinoma screening, mainly breast self-examination and mammography, were predicted to improve as a primary outcome. The results showed that behavioral intent to do breast self-examination (Baseline=103.77, 4 months; 121.50, 8 months; 97.12, 12 months; 119.19) and mammography (Baseline=103.77, 4 months; 121.50, 8 months; 97.12, 12 months; 119.19) improved in the intervention group, however, certain level of change was also noticed in the control group.
**Order of Authors:**	Rojana Dhakal, M.Sc N(Obstetrics and Gynecology)
	Chiranjivi Adhikari, Master in Public Health
	Prabha Karki, M.Sc.N(Psychiatric)
	Nirmala Neupane, M.Sc N(Obstetrics and Gynecology)
	Pooja Bhandari, M.Sc N(Obstetrics and Gynecology)
	Aditi Gurung, M.Sc.N (Paediatric)
	Nisha Shrestha, M.Sc.N(Psychiatric)
	Nandaram Gahatraj, Master in Public Health
	Niranjan Shrestha, PhD (Statistics)
	Niranjan Koirala, PhD
	Govind Subedi, PhD
**Additional Information:**	

Powered by Editorial Manager® and ProduXion Manager® from Aries Systems Corporation

Question	Response
**Financial Disclosure**<br><br>Enter a financial disclosure statement that describes the sources of funding for the work included in this submission. Review the submission guidelines for detailed requirements. View published research articles from *PLOS ONE* for specific examples.<br><br>This statement is required for submission and **will appear in the published article** if the submission is accepted. Please make sure it is accurate.<br><br>**Unfunded studies**<br>Enter: *The author(s) received no specific funding for this work.*<br><br>**Funded studies**<br>Enter a statement with the following details:<br>• Initials of the authors who received each award<br>• Grant numbers awarded to each author<br>• The full name of each funder<br>• URL of each funder website<br>• Did the sponsors or funders play any role in the study design, data collection and analysis, decision to publish, or preparation of the manuscript?<br>• **NO** - Include this sentence at the end of your statement: *The funders had no role in study design, data collection and analysis, decision to publish, or preparation of the manuscript.*<br>• **YES** - Specify the role(s) played.<br><br>* typeset	This study received the faculty research grant from University Grants Commission, Nepal. The awarded fund goes to principal investigator official institute. Funders has no role in the study design, data collection, analysis, decision to publish, and preparation of manuscript.
**Competing Interests**<br><br>Use the instructions below to enter a competing interest statement for this submission. On behalf of all authors, disclose any competing interests that could be perceived to bias this work—acknowledging all financial support and any other relevant financial or non-financial competing interests.	There was no competing interest exists among authors.

This statement is required for submission and **will appear in the published article** if the submission is accepted. Please make sure it is accurate and that any funding sources listed in your Funding Information later in the submission form are also declared in your Financial Disclosure statement.<br><br>View published research articles from *PLOS ONE* for specific examples.<br><br>**NO authors have competing interests**<br><br>Enter: *The authors have declared that no competing interests exist.*<br><br>**Authors with competing interests**<br><br>Enter competing interest details beginning with this statement:<br><br>*I have read the journal's policy and the authors of this manuscript have the following competing interests: [insert competing interests here]*<br><br>* typeset	
**Ethics Statement**<br><br>Enter an ethics statement for this submission. This statement is required if the study involved:<br><br>• Human participants<br>• Human specimens or tissue<br>• Vertebrate animals or cephalopods<br>• Vertebrate embryos or tissues<br>• Field research<br><br>Write "N/A" if the submission does not require an ethics statement.<br><br>General guidance is provided below. Consult the submission guidelines for detailed instructions. **Make sure that all information entered here is included in the Methods section of the manuscript.**	As this study required human subjects, the study was approved by the institutional review committee at Pokhara University (28/75-76). The study also received approval from the respective wards (94, 2751/76/77). In addition to this, informed written consent was obtained from the research participants, those who could not sign their consent, those participants signed consent by using thumb prints after explaining the details of the data collectors or relatives of participants.

Powered by Editorial Manager® and ProduXion Manager® from Aries Systems Corporation

**Format for specific study types**<br><br>**Human Subject Research (involving human participants and/or tissue)**<br>• Give the name of the institutional review board or ethics committee that approved the study<br>• Include the approval number and/or a statement indicating approval of this research<br>• Indicate the form of consent obtained (written/oral) or the reason that consent was not obtained (e.g. the data were analyzed anonymously)<br><br>**Animal Research (involving vertebrate animals, embryos or tissues)**<br>• Provide the name of the Institutional Animal Care and Use Committee (IACUC) or other relevant ethics board that reviewed the study protocol, and indicate whether they approved this research or granted a formal waiver of ethical approval<br>• Include an approval number if one was obtained<br>• If the study involved *non-human primates*, add *additional details* about animal welfare and steps taken to ameliorate suffering<br>• If anesthesia, euthanasia, or any kind of animal sacrifice is part of the study, include briefly which substances and/or methods were applied<br><br>**Field Research**<br><br>Include the following details if this study involves the collection of plant, animal, or other materials from a natural setting:<br>• Field permit number<br>• Name of the institution or relevant body that granted permission	
**Data Availability**<br><br>Authors are required to make all data underlying the findings described fully available, without restriction, and from the time of publication. PLOS allows rare exceptions to address legal and ethical concerns. See the PLOS Data Policy and FAQ for detailed information.	Yes - all data are fully available without restriction

A Data Availability Statement describing where the data can be found is required at submission. Your answers to this question constitute the Data Availability Statement and **will be published in the article**, if accepted.<br><br>**Important:** Stating 'data available on request from the author' is not sufficient. If your data are only available upon request, select 'No' for the first question and explain your exceptional situation in the text box.<br><br>Do the authors confirm that all data underlying the findings described in their manuscript are fully available without restriction?	
**Describe where the data may be found in full sentences. If you are copying our sample text, replace any instances of XXX with the appropriate details.**<br><br>• If the data are **held or will be held in a public repository**, include URLs, accession numbers or DOIs. If this information will only be available after acceptance, indicate this by ticking the box below. For example: *All XXX files are available from the XXX database (accession number(s) XXX, XXX.*).<br>• If the data are all contained **within the manuscript and/or Supporting Information files**, enter the following: *All relevant data are within the manuscript and its Supporting Information files.*<br>• If neither of these applies but you are able to provide **details of access elsewhere**, with or without limitations, please do so. For example:<br><br>*Data cannot be shared publicly because of [XXX]. Data are available from the XXX Institutional Data Access / Ethics Committee (contact via XXX) for researchers who meet the criteria for access to confidential data.*<br><br>*The data underlying the results presented in the study are available*	The dataset supporting the conclusions of this study is included within the article. Individual data used in this study is available upon request to the corresponding authors without any restrictions.

from (include the name of the third party and contact information or URL).<br>• This text is appropriate if the data are owned by a third party and authors do not have permission to share the data.<br><br>* typeset	
Additional data availability information:	Tick here if your circumstances are not covered by the questions above and you need the journal’s help to make your data available.; Tick here if the URLs/accession numbers/DOIs will be available only after acceptance of the manuscript for publication so that we can ensure their inclusion before publication.

Powered by Editorial Manager® and ProduXion Manager® from Aries Systems Corporation

Manuscript

Click here to access/download;Manuscript;Intention to perfrom BSE and Mammography_final.docx

The effect of educational intervention on intention to perform breast self-examination and mammography among women of Pokhara Nepal: application of theory of planned behavior

Rojana Dhakal[1*], Chiranjivi Adhikari [2,3], Prabha karki[1], Nirmala Neupane[1], Pooja Bhandari[1], Aditi Gurung[1], Nisha Shrestha[4], Nandaram Gahatraj[2], Niranjan Shrestha[2], Nirajan Koirala[5], Dr Govind Subedi[6]

1. Nursing Program, School of Health and Allied Sciences, Pokhara University, Pokhara, Gandaki Province, Nepal
2. Public Health Program, School of Health and Allied Sciences, Pokhara University, Pokhara, Gandaki Province, Nepal
3. Indian Institute of Public Health Gandhinagar (IIPHG), Gujarat-382042, India
4. Nursing Program, Pokhara Nursing Campus, Institute of Medicine, Tribhuvan University, Pokhara, Gandaki province, Nepal
5. Dr. Koirala Research Institute for Biotechnology and Biodiversity, Kathmandu 44600, Nepal
6. Central Department of Population Studies, Tribhuvan University, Kritipur, Bagmati Province, Nepal

Rojana Dhakal
ROLES Conceptualization, project administration, data management, Formal analysis, funding achievement, inquiry, Methodological review, Intervention design and finalization , writing- original draft, review and editing

Chiranjivi Adhikari
ROLES: Conceptualization, Methodology, Instrument development, Analysis, draft review and editing, final manuscript

Prabha karki
ROLES: Instrument development, data collection, intervention plan, writing review: draft and editing, final manuscript

Nirmala Neupane
ROLES: Instrument development, Intervention strategy, writing: reviewing and editing, final manuscript

Pooja Bhandari
ROLES: Instrument development, Intervention design, writing: reviewing and editing, final manuscript

Aditi Gurung
ROLES: Instrument development, Intervention design, writing original draft, final manuscript

Nisha Shrestha
ROLES: Instrument development, Intervention design, writing: reviewing and editing, final manuscript

Nandaram Gahatraj
ROLES: Instrument development, data management, formal analysis, software, final manuscript

Niranjan Shrestha
ROLES: Instrument development, formal analysis, Software, final manuscript
Niranjan Koirala
ROLES: Supervision and Guidance, writing: review editing and finalizing, final manuscript
Govind Subedi
ROLES: Methodology, Investigation, Supervision and Guidance, writing: review and editing, final manuscript

* rojanadkl@pu.edu.np/rojanabuddhi2@gmail.com
Abstract

Breast malignancy is the most frequent carcinoma among females across the world. Early diagnosis of breast cancer by breast health awareness and self-examination with mammography screening is a highly feasible and useful technique in poor resource countries. The primary objective of this study was to assess how an educational intervention affected women's intention to do breast self-examination and mammography screening in selected wards in Pokhara metrocity of Nepal. A non-randomized intervention was employed among 360 women (180 in intervention and 180 in control group) of 40-75 years. First, wards were randomly assigned in the intervention and control groups, then participants who satisfied inclusion criteria were purposefully chosen. Study participants in the intervention group were given a one-hour breast cancer screening related session. Also, respondents in the comparison group were given the similar educational session at the completion of the study. Relevant data was obtained at the baseline and, 4, 8 and 12 months of intervention. The intention to undertake breast carcinoma screening, mainly breast self-examination and mammography, were predicted to improve as a primary outcome. The results showed that behavioral intent to do breast self-examination (Baseline=103.77, 4 months; 121.50, 8 months; 97.12, 12 months; 119.19) and mammography (Baseline=103.77, 4 months; 121.50, 8 months; 97.12, 12 months; 119.19) improved in the intervention group, however, certain level of change was also noticed in the control group.

Key words: Breast self-examination, intention, mammography, theory of planned behavior

Introduction

Globally, breast tumor is the topmost malignancy in females (1,2). Breast carcinoma affects one out of every four women and is responsible for one in every six cancer deaths, placing it top in terms of incidence (159 of 185) and mortality (110 nations)(3). In 2020, breast cancer comprised 24.5% of all cases and 15.5% of all deaths among females in the globe (3). In the year 2020, Asia reported a rate of 10.8% of breast cancer cases and 6.0% of fatalities (4). Breast cancer is the most common third cancer in Nepal, with an incidence rate of 9.6% among total cancer cases and mortality rate of 7.7% in 2020(5). According to projections, the number of breast

tumor cases and fatalities in Nepal will reach 3579 thousand and 2014 thousand respectively by 2040(6).

Breast cancer early screening is an effective strategy that early identifies the tumor that contributes to the early treatment and enhances survivorship. Breast self-Examination (BSE), clinical breast exam (CBE) and mammogram x-ray are some of the maximum used screening tests. This test aids in the early detection of cases that are still curable(7). However, the American Cancer Society (ACS) no longer recommends BSE as a routine schedule or diagnostic tools, but it can be used in areas or countries where mammography screening is not available at the population level. BSE can be begun as early as the age of 20 to examine and feel one's breast and note any abnormalities(8,9). Women should begin regular screening starting at age 45 years old. Women must have the chance to begin a yearly screening test between the ages of 40 and 44 years old. Women aged 45-54 years old should be screened annually and Women aged 55 years and older should be tested every two years with mammography screening should continue as long as their overall well-being is satisfactory and they have a lifetime expectancy of ten years or more (10,11).

Cancer diagnostics and treatment facilities are exceedingly limited in low - resource countries like Nepal, Pakistan, Iraq, and Mongolia(12). In Nepal, there is no national or regional breast cancer screening program, and national breast cancer management protocols are still in the infancy stage(13). BSE, CBE, Mammography, Fine Needle Aspiration and Breast Ultrasound are the most widely utilized breast cancer diagnostic techniques in Nepal(14,15). Early identification of cancer cases and raising awareness about the need for screening procedures minimize the number of people who go to the hospital late for screening and treatment because they are unaware of screening measures(16,17). Though BSE is considered as an effective tool for detecting breast cancer in its early stages, research conducted in Butwal Sub-metropolitan city of Nepal found that only 31% had ever heard of BSE and only 19.2% had ever practiced it. This shows a huge gap in knowledge and practice of BSE among Nepalese women(18). According to research study, cancer treatment is improving in Nepal, but there is still lack of awareness about the symptoms, early diagnosis, and screening tests for breast cancer (19).

Intentions have a tremendous influence on behavior. In a study by Norman(20) intention was the sole predictor of BSE. Theory of planned behavior (TPB) is one of several health behavior theories that have been developed to predict health behavior. It focuses on women's attitude and beliefs (21). Attitude, subjective norms, and perceived behavioral controls are the three components of this theory(22). A study done in Kathmandu Nepal, found out of 500 women 3.4%, 7.2% and 14.4% of women had undergone mammography, clinical breast exam and breast self-examination respectively. If a woman believes she is at risk of breast cancer, she is more likely to undertake

screening measures. The researchers also recommend designing a culturally relevant intervention to improve screening intentions (23). Further, In Iranian research, Jeihooni and colleagues discovered that an educational intervention based on TPB increased the intention to get mammography screening for breast cancer(24). To date, there has been very little study on the effectiveness of educational intervention in determining women's desire to go for breast cancer screening. Hence, the present study's main aim is to find out the effect of educational intervention on the intention to engage in breast cancer screening methods such as BSE and mammography.

Methods and Materials

Study design and setting

A quasi-experimental controlled time series study with a control group design was conducted to assess the effectiveness of educational intervention on the intent to perform breast cancer screening among women in Pokhara, Nepal. The field work was conducted in Pokhara Metropolitan 30 and 33 wards, in Nepal's Gandaki province from May 2018 to March 2020. A total sample of 360 women (180 in the interventional and 180 in the control group) aged between 40-75 years with no history of benign or malignant breast disease were studied. Respondents were selected by using purposive sampling. The sample size was determined using the Open Epi sample size calculator for a cross sectional study with a power of 80%, and a confidence level of 95%, with an assumption of study from mammography utilization(25), percentage of unexposed and exposed with outcome 27% and 43% respectively. The calculated sample size was 154 in the exposed group and 154 in the non-exposed group. The total calculated minimum sample size was 308. Considering 16% drop out rate, the targeted sample size was increased to 360.

Data collection instruments

The questionnaire was developed by the researchers using the theory of planned behavior principles, considerable reviews, and expert comments. The questionnaire was prepared in the English language and then translated into the Nepali language and translated back to English. An oncologist, psychiatric nurse, gynecologist, nurse midwife, and health promotion specialist made up the expert panel. Formatted research was conducted to develop the tools. The instrument was pretested to 40 women living in ward number 10 of Pokhara. Some modifications were made to make it understandable for women. Each construct had a Cronbach's alpha greater than 0.7. Further, the design educational set was validated by experts, which comprises both theoretical and practical lessons. Based on the literature, intention scales including a total of 16 items for BSE and 18 items for mammography screening items were created. A five-point Likert scale was used to measure the intention of doing BSE and Mammography. On a scale of one to five, one represents "strongly disagree" and five represents "strongly agree" and vice versa in negatively meaning items (26–31).

Exposure variables and assessment

Attitude, perceived behavioral control, subjective norms and behavioral intention (constructs of theory of planned behavior)

Icek Ajzen (1985, 1991) created the Theory of Planned Behavior (TPB) as a comprehensive paradigm for understanding, predicting, and explaining behaviors. Behavioral intentions are instantaneously decided by behavioral intent, according to this theory, and three elements influence behavioral intentions: attitude, subjective norms, and perceived behavioral control(27,29). Attitude of behavior consists of behavioral beliefs and outcome behavior, Subjective norms of behavior consists of normative beliefs and motivation to comply, while perceived behavior control includes control belief strengths and power of control factors. Each construct was multiplied by its sub constructs independently, and the overall score of the main constructs was computed. After that, the total score for behavioral intention was determined by adding all three key constructs: attitude, subjective norms, and perceived behavior control and the final score for behavioral intention was calculated. Favorable attitudes, greater compatibility with referents, good or higher perceived control behavior and high desire to perform activity were all associated with a higher score.

Outcome variables

The intention to go for monthly breast self-examination and mammography every two years was the primary result of this research. The theory of planned construction of behavioral intention based on score cut-off points. The cut-off points were assigned as the median value at baseline data.

Data collection

Data was collected through face to-face interviews at baseline, 4 months, 8 months, and 12 months periods in both the interventional and control groups. The educational intervention consists of a one-hour session based on TPB. Every time the same tool was used to collect the information, the intervention group was given a repeated intervention in a time frame. Breast anatomy, breast cancer facts, signs and symptoms, risk factors, screening measures, treatment, preventive measures, BSE and Mammography were all discussed throughout the educational session. The Breast Self-Examination technique was taught and demonstrated. The intervention group was educated using a variety of methods, including lectures, discussion, and demonstrations. The American Cancer Society guidelines(10,32) , WHO(33-35) and International Agency for Research on Cancer(36) recommendations were used to create the lecture and demonstration guides. Participants demonstrated the BSE techniques (explain and show) and palpation (demonstrate in silicon model).

Ethics statement

The Institutional Review Committee (IRC number 28-75-76) of Pokhara University granted ethical approval. A formal permission from respected wards of the Pokhara Metropolitan was obtained. The participants gave their oral and written consent. The comparison group provided the same informative session at the end of the research. Privacy and confidentiality were maintained. Women participated voluntarily in the study and their names were not used in the study reports. Also, they were informed about the publication of the study results. At the conclusion of the research, the control group offered the identical instructional session.

Data analysis

Epi-data Version 3.1 was used to enter data, which was exported to SPSS version 25 for analysis. Descriptive and inferential statistics were calculated based on the nature of the variables and objectives of the study. The efficacy of the intervention was assessed using independent and paired t tests. Tables were used to represent the information. Statistical significance was defined as a p value of 0.05.

Results

Out of 360 women, in both the interventional and control groups, the largest number of women were in the age bracket of 40-50 years. **Table 1** summarizes the baseline aspects of the women included in the study. The average age in the interventional and control groups was 49. Hinduism was practiced by the vast majority of women. In the interventional and control groups, respectively, 25.6 percent and 30.6 percent of individuals were illiterate. In both groups, the number of individuals living in nuclear and joint households was about equal. Chi-square tests confirm that there were no significant variations between the two groups in any of the demographic and reproductive variables.

Table 2 demonstrates the BSE intention based on the TPB. At the start of the study, 6.7% of women in the intervention group and 2.8% of women in the control group expressed favorable or positive attitudes towards performing BSE. However, in the experimental group's subsequent posttests at 4, 8, and 12 months, favorable attitudes towards conducting BSE were 21.1%, 18.9%, and 22.8 %, respectively, following intervention. In addition, some changes were found in the control group without educational intervention, with 16.1% of individuals intending to conduct BSE 12 months of the pretest. After the educational intervention, the intervention group's compatibility with referents improved in posttest 1 (4 months) and posttest 2 (8 months). However, there were unexpected improvements in the control group, with compatibility with referents increasing to 78.9% at posttest3 from 35.6 percent at baseline. Surprisingly, both groups had higher levels of perceived behavior control and behavioral intention to execute BSE.

Table 3 illustrates the description of mammography intentions among the intervention and control groups before and after educational intervention. The results showed that 47.8% and 43.3% of women from intervention and control groups respectively had a favorable attitude towards performing

mammography screening in pretest. After intervention, the experimental group had a higher positive score of 77.2% at 4 months, 84.4% in the 8 months and 83.0% in the 12 months. In contrast, the control group participants had also increased positive attitudes towards performing mammography, but less favorable scores than the intervention group, 43.3% from baseline to 60.8% in the 12months. Further, there was 22.8% of women intended to perform mammography screening, in 4 months in the control groups. Next, with regards to subjective norms in the pretest, 55.0% of intervention and 44.4% of control group participants were compatible with referents. The participants in both groups had increased compatibility with the appropriate reference group for their intention to perform mammography. Surprisingly, it was slightly higher among the control group (75% and 77.2% respectively) at 8 months and 12 months compared to the intervention group (70% and 64.8% respectively).

Regarding Perceived Behavioral Control, 91.7% and 93.3% of the intervention and control groups respectively had poor control over getting screening for breast cancer by mammography. In addition to this, intervention group participants after the first intervention, their control was slightly increased, 21.7% had good perceived control, but still 80% of participants had poor perceived control in subsequent posttest3 to get screened through mammography. In the control group, a majority of 90% of participants had poor perceived control in posttest3, as only 10.0% of participants had good perceived control. A similar number of women from both groups had the intention of performing mammography in the future in pretest. Further, there was fluctuation in the intention to perform mammography. High intention was found in subsequent posttest 1 (87.2%) and dropped in posttest 2 (37.2%) and again increased in posttest 3 (83.6%). In the control group, it was slightly increased in subsequent posttest 1 (44.4%) to Posttest 3 (58.3%), but it was lower than in the intervention group.

Table 4

revealed information related to the effectiveness of educational intervention with regards to performing BSE. The independent t test showed that there were no significant differences observed before intervention in average scores of attitude (p=0.59), perceived behavioral control (0.33), and behavioral intention (0.08) in the experimental and control groups except in subjective norms (0.02). After educational intervention, significant differences were observed in the attitude towards performing BSE in the intervention group compared to control groups. With regards to subjective norms, both groups are compatible with referents as shown by increasing mean scores. Further, results showed that after intervention at 4 months and 12 months, significant differences were found between the intervention and control groups except in the 8 months regards referents with peers. Next, regarding perceived behavior control (PBC), there was a difference found after intervention (at 4 months) between the intervention and control groups. Then, there was no significant change observed even after the intervention was given in the control factors in the experimental groups. Furthermore, there was no difference in behavioral intention to

perform BSE in the pretest between the two groups and similar results were obtained in 12 months. The main difference was observed between the groups at 4 and 8 months.

With regards to mammography, the independent t test revealed that there were significant differences observed between the intervention and control groups in all the independent pairs regarding attitudes. Accordingly, the results of the independent t test indicated that, with regards to subjective norms, after the first intervention, there were statistically significant differences observed ($p=0.002$), participants were compatible with referents to perform mammography. After then, no statistically significant variations in outcomes were found between the intervention and control groups. Next, there was significant enhancement in the mean score of perceived behavior control after the first and third intervention. Unfortunately, there was no significant enhancement in the mean value in the intervention group after the second intervention. Furthermore, there was enhancement of behavioral intention mean score to get screened by mammography after educational intervention in both groups, but the experimental cluster had a lower value level than the comparison groups. However, the p value was less than 0.05 in all the independent pairs of behavioral intentions to have mammography.

Table 5

Table 5 showed the changes in the intention to perform breast self-examination from baseline between groups, after intervention following at 4, 8 and 12 months. Within the experimental group's attitude, subjective norms, perceived behavioral control and intent to perform BSE all improved significantly from baseline to different endpoints. However, in the case of subjective norms there were no changes significantly observed in the 8 months endpoint, for capacity of performing BSE, the changes not observed at 8 and 12 months endpoint. Further, intent to perform BSE changes observed only at 4 months endpoint.

Table 6 showed the changes in the intention to perform mammography from baseline between groups, after intervention following at 4, 8 and 12 months. Within the experimental group's attitude to undergoing mammography all improved significantly from baseline to three different endpoints. Further, subjective norms, perceived behavioral control and behavioral intent to undergo mammogram significant changes were not observed in the 8, 12 months endpoint of first intervention, only significant changes observed at 4 months after first intervention.

Discussion

The purpose of this study was to see if women aged 40 to 75 years will be willing to get BSE and mammography in the future based on the TPB construction. The attitude towards behavior is individual. It varies in

how beneficial, pleasurable or pleasant they believe behavior to be(27). According to TPB, attitude, subjective norms, and perceived behavioral control can all predict the intention to participate in various types of activity which brings considerable changes in behavior and variables those related to salient behavioral, normative, and control factors(37). Moreover, whereas both the interventional and control groups had identical attitudes regarding BSE at the start of the study, the experimental group's attitude toward BSE rose four months following the educational intervention. Differences amongst the intervention and control groups were also evident in present study. The control group, on the other hand, had a very favorable attitude towards mammography and exhibited some evidence of progressive change. This might be attributed to numerous visits to control groups, where the same questions were given each time, which could raise issues or may be over reported by participants, which could lead to certain changes. People develop favorable or positive mindsets as an outcome of educational instruction. This finding is similar with prior research, suggesting that intervention work enhance the attitudes scores Ali Khani Jeihooni et.al (38), Norman et.al (31),Steadman et.al (39) indicated the changed towards more favorable attitudes. Further, a favorable attitude was achieved in the experimental group by describing the circumstances of breast cancer, asking and answering questions, and having conversations to identify the beliefs and behavior outcomes in order to modify the negative attitudes and build the positive attitudes (38). In the present study, attitude towards performing BSE was significantly different between the experimental and control groups after educational intervention.

Next, the experimental group's average subjective norm score did not significantly improve after educational intervention. At 8 months, both groups had a comparable number of women who thought they were compatible with referents. However, mean changes were observed, on the other hand, revealed significant differences at 12 month endpoints. The education session of the present study was not held for spouses, children and family members but discussed how participants had been influenced by their peers and their drive to comply with their advice (22,27,29,39). The findings of the present study corroborate the study by Yamile et.al (40)in which the importance of subjective norms to perform breast cancer screening was highlighted.

The term "perceived behavioral control" (PBC) refers to a person's views about their ability to engage in a certain activity. It was stated that when people believe they have control over their conduct, they get motivated and do such activities even in difficult conditions (37,41). In accordance with earlier research (31), one of the important factor of TPB constructs to predict the BSE intentions and behavior is perceived control. According to the outcomes of this study, the intervention group's PBC toward executing BSE changed significantly after intervention. But, in the 8 months period

and 12 months of baseline, however, no significant differences were seen between the intervention and control groups. Findings of study said that, by educating and increasing women's abilities to conduct BSE will greatly enhance their behavior. In current study, lecturing and discussing the issue, showing pictures and conducting BSE in silicone model improved the intervention group's perceived behavior control (42–45).

The findings of this investigation revealed that the experimental and control groups had nearly identical intentions of doing BSE. The first intervention was more successful than the second as evidenced by p value and mean changes. Participants in the experimental group (55.0%) and the control group (43.9%) showed a strong intention to conduct behavior. The experimental group's desire to perform BSE rose after educational intervention (83.3 percent), (87.2 percent), and (73.1 percent) of women reported a high intention to do BSE in the first, second, and third post-intervention follow ups, respectively. In the control group, however, (37.8%) at the 1st follow up, (81.7%) at the 2nd follow up, and (80.6%) at the 3rd follow up, had high intention of doing BSE in the future. The theory of planned behavior is based on the belief that when an individual has a strong desire to engage in a behavior, the likelihood of engaging in that behavior becomes more feasible, higher, and successful (41,46). A person's intention to act is the most important factor in performing healthy behavior, but whether that intention can modify one's actions is still debatable (41). The findings of the present study were supported by the studies of Shelley N. Aikman on the influence of instruction point of view on breast self-examination self-efficacy (47), and a study on breast cancer in Zahedan showed that after educational intervention, mean scores of knowledge, attitude, perceived behavior control, behavioral intention, and adopting screening behaviors improved in the intervention group compared to control (48),also supported by study by Dewi et.al(41).

The present study found that nearly half of women in the intervention group (47.8%) and the control group (43.3%) had a favorable attitude towards performing mammography at the baseline. The experimental group's attitude towards performing mammography improved after 4, 8, and 12 months of intervention. The control group's attitude remained positive with minor changes. The present study showed that the intervention was successful in changing people's attitudes towards more favorable. The findings were consistent with the study published by Ali et.al regarding mammography (38), the study by Barbara K Rimer (49) and a Swedish study (50). The present study observed the improvement of average score of the subjective norms after informative session. Moreover, study said that family/friend/spouse recommendations increase perceived mammography norms, which affects intention and uses of mammography (40), subjective norms worked as an motivational factors by following important people in life (44). In the current study, the intervention group's compatibility with referents was observed in each subsequent follow up at 4, 8 and 12 months,

whereas similar changes occurred in the control group without any intervention, almost comparable with the study by Sargezi (48) where subjective norms did not change. In the present study, spouses, family members, and children are the most significant peoples who have a role in increasing compatible behavior, similar to a study of china (51) and another study from Isfahan Iran (52).

The current study found that relatively few participants (8.3%) and (6.7%) of individuals in the intervention and control groups believing they had control over their behavior in the pretest, respectively. Furthermore, women in the intervention group (21.3%), (10.6%) and (19.9%) have good control over performing mammography, whereas women in the control group (6.1%), (11.1%), and (10%) have perceived control over mammography screening at 4, 8 and 12 months respectively. There was a significant difference observed (p=0.001) in the mean score in all endpoints except 8 months.

Previous research on mammography screening and perceived control in Iranian women found that while mammography screening is not entirely under the control of the individual, psychological barriers such as embarrassment, fear of breast cancer diagnosis, family support, exposure to radiation, painful technique, cost, and long waits in public mammography centers must be addressed in order to make mammography more accessible (53,54). The findings of a study on the intention to maintain mammography adherence show that women who have a stronger sense of control are more likely to obtain a mammogram, even if no intervention is provided (55). A study of Iranian women regarding mammography screening (38), a study from Malaysia related to breast cancer screening (43) and study of women 40 years and above , Isfahan, Iran (56) all validated the improvement in perceived control following educational intervention.

Regards to Behavioral Intention, to get screened for breast cancer by mammography, indicated a change in mean score and the number of women in line with a high intention to perform mammography after intervention. However, some changes were seen in the control group as well, although the intervention group contained a substantial proportion of women who wanted to have mammography. There were significant differences observed in the intervention and control groups related to behavioral intention towards mammography after educational intervention. There were significant changes in behavioral intentions toward mammography following educational intervention between the intervention and control groups. Several research on breast and mammography screening supports the conclusion that educational intervention improved mean screening results and was effective (25,38,43,45,56,57).

The strength of this study is that, first, the TPB model has been used as a framework in order to implement educational interventions. Second, repeated measures of intervention were implemented among the women. Third, the TPB model was used to evaluate the intention of doing breast self-examination and mammography. The present study is only for women between the ages of 40-75 years. Second, the study didn't allow random assignment of participants, only the study area was chosen at random. Third, the study relied on a self-developed intention tool based on TPB guidelines for question construction.

Conclusion

Overall, the results of the study showed that adopting an educational intervention based on the theory of planned behavior enhanced the intention to do breast self-examination and mammography. Women are more likely to have high intent to do screening behaviors if they are given appropriate knowledge, confidence in doing screening behaviors, support from referents, and a good attitude. In the current study, a single intervention produces a more significant change in behavior than repeated interventions. Educational intervention based on the theory of planned behavior promotes the intent to do breast self-examination and mammography. It is suggested that we use this theory to improve behaviors regarding breast cancer screening. Also, attitude and perceived behavioral control are the main constructs that determine behavior, so it is recommended that individuals study the constructs to change behavior.

Acknowledgements
We would like to express our gratitude to the University Grants Commission (UGC-Nepal) of Nepal for awarding faculty research grants (FRG-73/74-HS-15) to carry out this research. In addition to this, we would like to express the deepest gratitude for study participants and B.Sc. Nursing 6th and 7th batch students of School of Health and Allied Sciences, Pokhara University for enormous support during the phase of data collection and intervention. RD, NN, PB, AG, NS received the grants to carry out the research from UGC-Nepal.
Conflict of Interests
There is no competing interest between the authors and with the granter.

References

1. Breast cancer [Internet]. World Cancer Research Fund. 2018 [cited 2020 Nov 18]. Available from: https://www.wcrf.org/dietandcancer/breast-cancer

2. WHO | Breast cancer: prevention and control [Internet]. WHO. World Health Organization; [cited 2020 Nov 18]. Available from: http://www.who.int/cancer/detection/breastcancer/en/

3. Sung H, Ferlay J, Siegel RL, Laversanne M, Soerjomataram I, Jemal A, et al. Global Cancer Statistics 2020: GLOBOCAN Estimates of Incidence and Mortality Worldwide for 36 Cancers in 185 Countries. CA: A Cancer Journal for Clinicians. 2021;71(3):209–49.

4. IARC, WHO. Asia fact sheet breast cancer [Internet]. IARC, WHO; 2021 Mar p. 2. Available from: https://gco.iarc.fr/today/data/factsheets/populations/935-asia-fact-sheets.pdf

5. IARC, WHO. Population Fact Sheet Nepal [Internet]. International Agency for Research in Cancer, World Health Organization; 2021 p. 2. Available from: https://gco.iarc.fr/today/data/factsheets/populations/524-nepal-fact-sheets.pdf

6. WHO, IARC. Cancer Tomorrow [Internet]. 2020 [cited 2021 Aug 3]. Available from: https://gco.iarc.fr/tomorrow/en/dataviz/bars?types=0&sexes=2&mode=cancer&group_populations=0&multiple_populations=0&multiple_cancers=1&cancers=20&populations=524

7. GHODSI Z, HOJJATOLESLAMI S. Breast self examination and mammography in cancer screening: women health protective behavior. J Prev Med Hyg [Internet]. 2014 Jun [cited 2021 Jan 11];55(2):46–9. Available from: https://www.ncbi.nlm.nih.gov/pmc/articles/PMC4718330/

8. Dündar PE, Özmen D, Öztürk B, Haspolat G, Akyıldız F, Çoban S, et al. The knowledge and attitudes of breast self-examination and mammography in a group of women in a rural area in western Turkey. BMC Cancer [Internet]. 2006 Feb 24 [cited 2021 Jan 11];6(1):43. Available from: https://doi.org/10.1186/1471-2407-6-43

9. ACS Breast Cancer Early Detection Recommendations [Internet]. [cited 2021 Jan 11]. Available from: https://www.cancer.org/cancer/breast-cancer/screening-tests-and-early-detection/american-cancer-society-recommendations-for-the-early-detection-of-breast-cancer.html

10. Smith RA, Andrews KS, Brooks D, Fedewa SA, Manassaram-Baptiste D, Saslow D, et al. Cancer screening in the United States, 2018: A review of current American Cancer Society guidelines and current issues in cancer screening. CA: A Cancer Journal for Clinicians [Internet]. 2018 [cited 2021 Jan 4];68(4):297–316. Available from: https://acsjournals.onlinelibrary.wiley.com/doi/abs/10.3322/caac.21446

11. Smith RA, Andrews KS, Brooks D, Fedewa SA, Manassaram-Baptiste D, Saslow D, et al. Cancer screening in the United States, 2019: A review of current American Cancer Society guidelines and current issues in cancer screening. CA: A Cancer Journal for Clinicians [Internet]. 2019 [cited 2021 Jan 11];69(3):184–210. Available from: https://acsjournals.onlinelibrary.wiley.com/doi/abs/10.3322/caac.21557

12. Fan L, Goss PE, Strasser-Weippl K. Current Status and Future Projections of Breast Cancer in Asia. BRC [Internet]. 2015 [cited 2020 Dec 9];10(6):372–8. Available from: https://www.karger.com/Article/FullText/441818

13. Giri M, Giri M, Thapa RJ, Upreti B, Pariyar B. Breast Cancer in Nepal: Current status and future directions (Review). Biomedical Reports [Internet]. 2018 Apr 1 [cited 2020 Nov 8];8(4):325–9. Available from: http://www.spandidos-publications.com/10.3892/br.2018.1057/abstract

14. Singh YP, Sayami P. Management of Breast Cancer in Nepal. Journal of Nepal Medical Association [Internet]. 2009 Jul 1 [cited 2020 Nov 19];48(175). Available from: http://www.jnma.com.np/jnma/index.php/jnma/article/view/197

15. Giri M, Giri M, Thapa RJ, Upreti B, Pariyar B. Breast Cancer in Nepal: Current status and future directions. Biomed Rep [Internet]. 2018 Apr [cited 2020 Nov 19];8(4):325–9. Available from: https://www.ncbi.nlm.nih.gov/pmc/articles/PMC5838313/

16. Agarwal G, Ramakant P. Breast Cancer Care in India: The Current Scenario and the Challenges for the Future. Breast Care (Basel) [Internet]. 2008 Mar [cited 2021 Jan 11];3(1):21–7. Available from: https://www.ncbi.nlm.nih.gov/pmc/articles/PMC2931014/

17. da Costa Vieira RA, Biller G, Uemura G, Ruiz CA, Curado MP. Breast cancer screening in developing countries. Clinics (Sao Paulo) [Internet]. 2017 Apr [cited 2020 Aug 27];72(4):244–53. Available from: https://www.ncbi.nlm.nih.gov/pmc/articles/PMC5401614/

18. Marahatta SB, Sharma S. Knowledge and practice of breast self examination among women of reproductive age in Butwal Sub Metropolitan City. Journal of Manmohan Memorial Institute of Health Sciences [Internet]. 2018 Sep 22 [cited 2021 Jan 11];4(1):117–29. Available from: https://www.nepjol.info/index.php/JMMIHS/article/view/21149

19. Giri M, Giri M, Thapa RJ, Upreti B, Pariyar B. Breast Cancer in Nepal: Current status and future directions. Biomed Rep. 2018 Apr;8(4):325–9.

20. Norman P, Hoyle S. The Theory of Planned Behavior and Breast Self-Examination: Distinguishing Between Perceived Control and Self-Efficacy. Journal of Applied Social Psychology. 2004;34(4):694–708.

21. Lawal O, Murphy F, Hogg P, Nightingale J. Health Behavioural Theories and Their Application to Women's Participation in Mammography Screening. J Med Imaging Radiat Sci. 2017 Jun;48(2):122–7.

22. Mason TE, White KM. Applying an Extended Model of the Theory of Planned Behaviour to Breast Self-examination. J Health Psychol [Internet]. 2008 Oct 1 [cited 2020 Aug 27];13(7):946–55. Available from: https://doi.org/10.1177/1359105308095069

23. Bhandari D, Shibanuma A, Kiriya J, Hirachan S, Ong KIC, Jimba M. Factors associated with breast cancer screening intention in Kathmandu Valley, Nepal. PLoS One. 2021;16(1).

24. Khani Jeihooni A, Darvishi N, Harsini PA. The Effect of Educational Intervention Based on the Theory of Planned Behavior on Mammography Screening in Iranian Women. J Canc Educ. 2020 Apr 1;35(2):264–73.

25. Paskett E, Tatum C, Rushing J, Michielutte R, Bell R, Foley KL, et al. Randomized Trial of an Intervention to Improve Mammography Utilization Among a Triracial Rural Population of Women. J Natl Cancer Inst [Internet]. 2006 Sep 6 [cited 2020 Aug 27];98(17):1226–37. Available from: https://www.ncbi.nlm.nih.gov/pmc/articles/PMC4450352/

26. Mason TE, White KM. The Role of Behavioral, Normative and Control Beliefs in Breast Self-Examination. Women & Health [Internet]. 2008 Aug 30 [cited 2020 Sep 3];47(3):39–56. Available from: http://www.tandfonline.com/doi/abs/10.1080/03630240802132344

27. Ajzen I. The theory of planned behaviour: Reactions and reflections. Psychology & Health [Internet]. 2011 Sep 1 [cited 2020 Aug 27];26(9):1113–27. Available from: https://doi.org/10.1080/08870446.2011.613995

28. Ajzen I. CONSTRUCTING A THEORY OF PLANNED BEHAVIOR QUESTIONNAIRE. :8.

29. Kan MPH, Fabrigar LR. Theory of Planned Behavior. In: Zeigler-Hill V, Shackelford TK, editors. Encyclopedia of Personality and Individual Differences [Internet]. Cham: Springer International Publishing; 2017 [cited 2020 Sep 3]. p. 1–8. Available from: https://doi.org/10.1007/978-3-319-28099-8_1191-1

30. Knabe A. Applying Ajzen's Theory of Planned Behavior to a Study of Online Course Adoption in Public Relations Education. :256.

31. Norman P, Hoyle S. The Theory of Planned Behavior and Breast Self-Examination: Distinguishing Between Perceived Control and Self-Efficacy. Journal of Applied Social Psychology [Internet]. 2004 [cited 2020 Aug 27];34(4):694–708. Available from: https://onlinelibrary.wiley.com/doi/abs/10.1111/j.1559-1816.2004.tb02565.x

32. DeSantis C, Ma J, Bryan L, Jemal A. Breast cancer statistics, 2013. CA: A Cancer Journal for Clinicians [Internet]. 2014 [cited 2021 Jan 4];64(1):52–62. Available from: https://acsjournals.onlinelibrary.wiley.com/doi/abs/10.3322/caac.21203

33. WHO | Breast cancer: prevention and control [Internet]. WHO. World Health Organization; [cited 2020 Aug 27]. Available from: http://www.who.int/cancer/detection/breastcancer/en/

34. World Health Organization. WHO position paper on mammography screening. [Internet]. 2014 [cited 2020 Aug 27]. Available from: http://apps.who.int/iris/bitstream/10665/137339/1/9789241507936_eng.pdf?ua=1

35. Breast Self-Examination [Internet]. HealthLink BC. [cited 2020 Dec 14]. Available from: https://www.healthlinkbc.ca/health-topics/hw3791

36. Breast digital atlas - Breast self-Examination (BSE) [Internet]. [cited 2021 Jan 4]. Available from: https://screening.iarc.fr/breastselfexamination.php

37. Ajzen I. The theory of planned behavior. Organizational Behavior and Human Decision Processes [Internet]. 1991 Dec 1 [cited 2021 Jan 12];50(2):179–211. Available from: http://www.sciencedirect.com/science/article/pii/074959789190020T

38. Khani Jeihooni A, Darvishi N, Harsini PA. The Effect of Educational Intervention Based on the Theory of Planned Behavior on Mammography Screening in Iranian Women. J Canc Educ [Internet]. 2020 Apr 1 [cited 2021 Jan 4];35(2):264–73. Available from: https://doi.org/10.1007/s13187-018-1460-3

39. Steadman L, Rutter DR. Belief importance and the theory of planned behaviour: comparing modal and ranked modal beliefs in predicting attendance at breast screening. Br J Health Psychol. 2004 Nov;9(Pt 4):447–63.

40. Molina Y, Ornelas IJ, Doty SL, Bishop S, Beresford SAA, Coronado GD. Family/friend recommendations and mammography intentions: the roles of perceived mammography norms and support. Health Education Research [Internet]. 2015 Oct 1 [cited 2021 Jan 13];30(5):797–809. Available from: https://doi.org/10.1093/her/cyv040

41. Dewi TK, Zein RA. Predicting Intention Perform Breast Self-Examination: Application of the Theory of Reasoned Action. Asian Pacific Journal of Cancer Prevention [Internet]. 2017 Nov 1 [cited 2020 Sep 3];18(11):2945–52. Available from: http://journal.waocp.org/article_51456.html

42. Kisuya J, Wachira J, Busakhala N, Naanyu V, Chite AF, Omenge O, et al. Impact of an educational intervention on breast cancer knowledge in western Kenya. Health Educ Res. 2015 Oct;30(5):786–96.

43. Noman S, Shahar HK, Abdul Rahman H, Ismail S. Effectiveness of An Educational Intervention of Breast Cancer Screening Practices Uptake, Knowledge, and Beliefs among Yemeni Female School Teachers in Klang Valley, Malaysia: A Study Protocol for a Cluster-Randomized Controlled Trial. International Journal of Environmental Research and Public Health [Internet]. 2020 Jan [cited 2020 Dec 23];17(4):1167. Available from: https://www.mdpi.com/1660-4601/17/4/1167

44. Crombie K, Hancock K, Chang E, Vardanega L, Wonghongkul T, Chanakok A, et al. Breast screening education at Australian and Thai worksites: A comparison of program effectiveness. Contemporary Nurse [Internet]. 2005 Aug 1 [cited 2021 Jan 13];19(1–2):181–96. Available from: https://doi.org/10.5172/conu.19.1-2.181

45. Steele SK, Porche DJ. Testing the theory of planned behavior to predict mammography intention. Nursing Research. 2005;54(5):332–8.

46. Peyman N, Amani M, Esmaili H. The relationship between health literacy and constructs of theory of planned behavior and breast cancer screening tests performance among women referred to health care centers in Roshtkhar, 2015. Iranian Quarterly Journal of Breast Disease. 2016;9(3):60–9.

47. Aikman SN, Doyle-Portillo S, Verhaeghen P, Simmons N. The Effect of Instruction Point of View on Self-efficacy for Performing Breast Self-exams. American Journal of Health Education [Internet]. 2017 Feb 1 [cited 2020 Nov 7];48(1):1–10. Available from:

http://search.ebscohost.com/login.aspx?direct=true&db=ccm&AN=120458742&site=ehost-live&scope=site

48. Sargazi M, Mohseni M, Safar-Navade M, Iran-Pour A, Mirzaee M, Jahani Y. Effect of an Educational Intervention Based on the Theory of Planned Behavior on behaviors leading to early diagnosis of Breast Cancer among women referred to health care centers in Zahedan in 2013. :1.

49. Rimer BK, Halabi S, Skinner CS, Kaplan EB, Crawford Y, Samsa GP, et al. The short-term impact of tailored mammography decision-making interventions. Patient Education and Counseling [Internet]. 2001 Jun 1 [cited 2021 Jan 13];43(3):271–87. Available from: http://www.sciencedirect.com/science/article/pii/S0738399100001725

50. Zheng Mao, Nyström L, Jonsson H. Effectiveness of Population-Based Service Screening with Mammography for Women Aged 70-74 Years in Sweden. Cancer Epidemiology, Biomarkers & Prevention. 2020 Nov;29(11):2149–56.

51. Su X, Ma GX, Seals B, Tan Y, Hausman A. Breast Cancer Early Detection among Chinese Women in the Philadelphia Area. Journal of Women's Health [Internet]. 2006 Jun 1 [cited 2021 Jan 14];15(5):507–19. Available from: https://www.liebertpub.com/doi/abs/10.1089/jwh.2006.15.507

52. Mitra M, Ensiyeh N, Mohsen R, Firoozeh M, Gholamreza S. COMPARING THE EFFECTIVENESS OF THREE EDUCATIONAL INTERVENTIONS BASED ON HEALTH BELIEF MODEL ON MAMMOGRAPHY SCREENING BEHAVIORS IN WOMEN ABOVE 40 YEARS IN ISFAHAN, IRAN. 2013 Jan 1 [cited 2021 Jan 14];9(4):385–94. Available from: https://www.sid.ir/en/journal/ViewPaper.aspx?ID=342193

53. Khazir Z, Morowatisharifabad MA, Vaezi A, Enjezab B, Yari F, Fallahzadeh H. Perceived Behavioral Control in Mammography: A Qualitative Study of Iranian Women's Experiences [Internet]. International Journal of Cancer Management. 2019 [cited 2021 Jan 14]. Available from: https://sites.kowsarpub.com/ijcm/articles/90225.html#abstract

54. Godin G, Gagné C, Maziade J, Moreault L, Beaulieu D, Morel S. Breast cancer: The intention to have a mammography and a clinical breast examination - application of the theory of planned behavior. Psychology & Health [Internet]. 2001 Jul 1 [cited 2021 Jan 11];16(4):423–41. Available from: https://doi.org/10.1080/08870440108405517

55. O'Neill SC, Bowling JM, Brewer NT, Lipkus IM, Skinner CS, Strigo TS, et al. Intentions to Maintain Adherence to Mammography. J Womens Health (Larchmt) [Internet]. 2008 Sep [cited 2021 Jan 11];17(7):1133–41. Available from: https://www.ncbi.nlm.nih.gov/pmc/articles/PMC2575244/

56. Rezaeian M, Sharifirad G, Mostafavi F, Moodi M, Abbasi MH. The effects of breast cancer educational intervention on knowledge and health beliefs of women 40 years and older, Isfahan, Iran. J Educ Health Promot [Internet]. 2014 May 5 [cited 2020 Sep 16];3. Available from: https://www.ncbi.nlm.nih.gov/pmc/articles/PMC4089115/

57. Masso-Calderón AM, Meneses-Echávez JF, Correa-Bautista JE, Tovar-Cifuentes A, Alba-Ramírez PA, Charry-Ángel CE. Effects of an Educational Intervention on Breast Self-Examination, Breast Cancer Prevention-Related Knowledge, and Healthy Lifestyles in Scholars from a Low-Income

Area in Bogota, Colombia. J Canc Educ [Internet]. 2018 Jun 1 [cited 2020 Aug 27];33(3):673–9. Available from: https://doi.org/10.1007/s13187-016-1133-z

 Click here to access/download;Figure;Data table_plos one.docx

Table 1:

Baseline characteristics of participants (n=360)

Characteristics	Intervention group Frequency(percent)	Control group Frequency (percent)	P-value
**Age (years)**			
≤ 50 years	110 (61.1%)	115 (63.9%)	0.58
>50 years	70 (38.9%)	65 (36.1%)	
Mean ± SD	49.4±9.3	49.6±8.1	
**Religion**			
Hindu	162 (90.0%)	167 (92.8%)	0.49
Buddhist	16 (8.9%)	13 (7.2%)	
Muslim	1 (0.6%)	0 (0.0%)	
Christian	1 (0.6%)	0 (0.0%)	
**Marital Status**			
Married	168 (93.3%)	170 (94.4%)	0.82
Divorced/widow*	12 (6.7%)	10 (5.6)	
**Ethnicity**			
Brahmin	98 (54.4%)	101 (56.1%)	
Chhetri	18 (10.0%)	19 (10.6%)	
Dalit	12 (6.7%)	23 (12.8%)	0.14
Muslim	1 (0.6%)	0 (0.0%)	
Janjati	51 (28.3%)	37 (20.6%)	
**Education**			
Illiterate	46 (25.6%)	55 (30.6%)	
Can read or write	53 (29.4%)	57 (31.7%)	0.35
Literate	81 (45.0%)	68 (37.8%)	
**If Literate**			
SEE and below	62 (76.5%)	62 (91.2%)	
Higher Secondary	16 (19.8%)	5 (7.4%)	.05
Bachelor and above	3 (3.7%)	1 (1.5%)	
**Type of family**			
Nuclear	87 (48.3%)	91 (50.6%)	
Joint	86 (47.8%)	86 (47.8%)	0.43
Extended	7 (3.9%)	3 (1.7%)	

Table 2:

Descriptive statistics to perform breast self-examination based on the constructs of theory of planned behavior at different endpoints (n=360)

Categories	Intervention group				Control group			
	Baseline	4 month	8 month	12 month	Baseline	4 month	8 month	12 month
Attitude towards behavior								
Unfavorable (≤48)	168 (93.3%)	142 (78.9%)	146 (81.1%)	132 (77.2%)	175 (97.2%)	174 (96.7%)	153 (85%)	151 (83.9%)
Favorable (>48)	12 (6.7%)	38 (21.1%)	34 (18.9%)	39 (22.8%)	05 (2.8%)	06 (3.3%)	27 (15.0%)	29 (16.1%)
Subjective Norm (SN)								
Non Compatible (≤32)	80 (44.4%)	57 (31.7%)	53 (29.4%)	74 (43.3%)	116 (64.4%)	119 (66.1%)	38 (21.1%)	38 (21.1%)
Compatible (>32)	100 (55.6%)	123 (68.3%)	127 (70.6%)	97 (56.7%)	64 (35.6%)	61 (33.9%)	142 (78.9%)	142 (78.9%)
Perceived Behavioral Control (PBC)								
Poor (≤23)	80 (44.4%)	7 (3.9%)	6 (3.3)	10 (5.8%)	100 (55.6%)	101 (56.1%)	11 (6.1%)	11 (6.1%)
Good (>23)	100 (55.6%)	173 (96.1%)	174 (96.7%)	161 (94.2%)	80 (44.4%)	79 (43.9%)	169 (93.9%)	169 (93.9%)
Behavioral Intention (BI)								
Low (≤102)	81 (45.0%)	30 (16.7%)	23 (12.8%)	46 (26.9%)	101 (56.1%)	112 (62.2%)	33 (18.3%)	35 (19.4%)
High (>102)	99 (55.0%)	150 (83.3%)	157 (87.2%)	125 (73.1%)	79 (43.9%)	68 (37.8%)	147 (81.7%)	145 (80.6%)

Table 3:

Comparative frequency and percentage distribution of constructs at different endpoints of mammography intention (n=360)

Categories	Intervention group				Control group			
	Baseline	4 month	8 month	12 month	Baseline	4 month	8 month	12 month
				Attitude towards behavior				
Unfavorable (≤40)	94 (52.2%)	41 (22.8%)	28 (15.6%)	29 (17.0%)	102 (56.7%)	139 (77.2%)	53 (29.4%)	57 (31.7%)
Favorable (>40)	86 (47.8%)	139 (77.2%)	152 (84.4%)	142 (83.0%)	78 (43.3%)	41 (22.8%)	127 (70.6%)	123 (68.3%)
				Subjective Norm (SN)				
Non Compatible (≤31)	81 (45.0%)	50 (27.8%)	54 (30.0%)	61 (35.7%)	100 (55.6%)	90 (50.0%)	45 (25.0%)	41 (22.8%)
Compatible (>31)	99 (55.0%)	130 (72.2%)	126 (70.0%)	110 (64.8%)	80 (44.4%)	90 (50.0%)	135 (75.0%)	139 (77.2%)
				Perceived Behavioral Control (PBC)				
Poor (≤32)	165 (91.7%)	141 (78.3%)	161 (89.4%)	137 (80.1%)	168 (93.3%)	169 (93.9%)	160 (88.9%)	162 (90.0%)
Good (>32)	15 (8.3%)	39 (21.7%)	19 (10.6%)	34 (19.9%)	12 (6.7%)	11 (6.1%)	20 (11.1%)	18 (10.0%)
				Behavioral Intention (BI)				
Low (≤101)	82 (45.6%)	23 (12.8%)	113 (62.8%)	28 (16.4%)	100 (55.6%)	81 (45.0%)	142 (78.9%)	75 (41.7%)
High (>101)	98 (54.4%)	157 (87.2%)	67 (37.2%)	143 (83.6%)	80 (44.4%)	99 (55.0%)	38 (21.1%)	105 (58.3%)

Table 4:

Comparison of various constructs in mammography and breast-self-examination at different end-points post-interventions (n=360)

Variables	Time	Mammography			Breast self-examination		
		Intervention	Control	P value	Intervention	Control	P value
Attitude	Baseline	42.85(7.73)	41.93(6.15)	.217	44.17 (9.75)	43.65(8.60)	0.59
	4 month	49.20 (9.76)	41.36 (6.56)	.001*	50.37(9.30)	46.98 (7.01)	.001*
	8 month	51.73 (11.62)	44.06 (6.01)	.001*	50.24(8.50)	45.75 (7.99)	.001*
	12 month	57.39 (11.16)	43.93 (6.48)	.001*	52.16 (10.30)	45.78 (8.0)	.001*
Subjective Norms	Baseline	33.19(11.75)	32.36(10.80)	.048	34.14 (12.06)	31.42(10.31)	0.02*
	4 month	38.26 (13.04)	34.07 (11.82)	.002*	38.25(12.32)	31.78 (11.57)	.001*
	8 month	37.58 (14.30)	35.53 (6.59)	.082	38.81 (11.98)	38.26 (8.32)	.0613
	12 month	35.88 (13.58)	35.84 (6.75)	.969	34.82 (14.30)	38.22 (8.38)	.007*
Perceived Behavioral Control	Baseline	27.73(8.59)	27.57(6.15)	0.83	23.41(9.49)	22.54(7.54)	0.338
	4 month	34.03 (6.98)	31.28 (5.47)	.001*	32.85 (6.77)	20.54 (5.03)	.001*
	8 month	25.03 (4.32)	24.43 (4.02)	.178	31.70 (6.45)	32.12 (5.80)	0.509
	12 month	25.90 (5.18)	24.38 (3.90)	.002*	31.69 (6.93)	32.08 (5.76)	0.569
Behavioral Intention	Baseline	103.77(20.46)	101.87(13.96)	0.30	101.73(24.96)	97.62(19.73)	0.08
	4 month	121.50 (23.87)	106.72 (17.28)	.001*	121.48 (21.99)	99.32 (16.60)	0.001*
	8 month	97.12 (17.37)	92.04 (13.79)	.002*	120.76 (21.17)	116.15 (17.25)	0.024*

12 month	119.19 (21.68)	104.16 (11.38)	.001*	118.69 (24.13)	116.09 (17.40)	0.247

Table 5:

Mean changes in constructs of theory of planned behavior intent to perform breast self-examination by time and group (n=360)

Variables	Time	Intervention	Control	Difference (95% CI)	P value
Attitude	4 month	-6.20 (13.36)	-3.33 (8.16)	-2.86 (-5.16, -.57)	.015*
	8 month	-6.06 (11.90)	-2.10(11.06)	- 3.96 (-6.34, -1.58)	.001*
	12 month	-8.26 (14.82)	-2.13(10.93)	-6.13 (-8.86, -3.41)	.000*
Subjective Norms	4 month	-4.11(17.74)	-.36(10.96)	-3.74(-6.80,- .68)	.017*
	8 month	-4.67(16.07)	-6.84 (12.32)	2.17 (-.79, 5.14)	.151
	12 month	-1.15(17.86)	-6.80 (12.52)	5.64(2.41 – 8.87)	.001*
Perceived Behavioral Control	4 month	-9.43 (11.81)	2.0 (5.55)	- 11.43(-13.35,-9.52)	.001*
	8 month	-8.28 (10.49)	-9.58(9.27)	-1.29 (-.75, 3.34)	.216
	12 month	-8.50 (11.20)	-9.53(9.25)	-1.03 (-1.11, 3.18)	.345
Behavioral Intention	4 month	-19.75(33.20)	-1.70 (16.91)	-18.05(-23.51,-12.58)	.000*
	8 month	-19.02(29.32)	-18.52(23.58)	.50 (-6.01, 5.01)	.859
	12 month	-17.92(34.92)	-18.47(23.53)	.54(-5.67, 6.77)	.863

*significance, changes are calculated based on baseline value minus follow up time points, #n= 171 in experimental group at 12 month, p values are derived from independent samples t test

Table 6:

Mean changes in constructs of theory of planned behavior intent to perform mammography by time and group (n=360)

Variables	Time	Intervention	Control	Difference (95% CI)	P value
Attitude	4 month	-6.35 (11.49)	-.57 (8.29)	-6.92 (-9.00, -4.84)	.000*
	8 month	-8.88 (14.63)	-2.12 (8.68)	- 6.76 (-9.26, - 4.27)	.000*
	12 month	-14.69 (13.90)	-1.99(8.98)	-12.69 (-15.14, -10.25)	.000*
Subjective Norms	4 month	-5.06 (16.68)	-1.71(8.83)	-3.35 (-6.12,- .58)	.018*
	8 month	-4.38(17.33)	-3.16 (12.30)	-1.22 (-4.33, 1.89)	.441
	12 month	-2.95(17.56)	-3.47(12.65)	.51 (-2.68, 3.72)	.750
Perceived Behavioral Control	4 month	-6.30 (11.32)	-3.71 (7.23)	- 2.58 (-4.55,-.61)	.010*
	8 month	2.70 (9.37)	3.13 (7.75)	-.43 (-2.21, 1.35)	.633
	12 month	1.60 (10.68)	-3.18(7.42)	-1.57 (-3.49, .34)	.108
Behavioral Intention	4 month	-17.72(28.77)	- 4.85 (13.93)	-12.87(-17.55,-8.18)	.000*
	8 month	-6.65 (24.47)	- 9.83(17.88)	-3.17 (-7.62, 1.26)	.161
	12 month	-16.04(30.03)	-2.28(16.68)	-13.75 (-18.82, - 8.68)	.000*

*significance, changes are calculated based on baseline value minus follow up time points, n= 171 in experimental group at 12 month, p values are derived from independent samples t test,
