# Supplementary material for: Attitude sustains longer than subjective norm and perceived behavioral control: Results of breast cancer screening educational intervention
Source: PLoS One. 2023 Feb 10;18(2):e0281184. doi: 10.1371/journal.pone.0281184 (PMC9916560; doi:10.1371/journal.pone.0281184)
Supplement: Attachment — Submitted filename: Response to Reviewers.docx [file pone.0281184.s003.docx]

To:

The Editor in-Chief

Plos One

From:

Rojana Dhakal

27^th^ August , 2022

Dear Sir/Madam,

We would like to thank the reviewers for their generous comments on the manuscript entitled “***The effect of educational intervention based on the theory of planned behavior on intention to perform breast self-examination and mammography among women of Pokhara, Nepal: A quasi-experimental study”.*** *W*e have revised the manuscript by addressing all the concerns raised by both the editor and the reviewers. The responses along with the comments are described in detail in this response letter.

Regarding financial disclosure please make the following changes.

- This study received a faculty research grant from University Grants Commission Nepal (award number FRG-73/74-HS-15).

Regarding competing interest section please add following statements;

- The authors have declared that no competing interests exist.

Regarding availability of data;

- The data are available in the supporting information file.

Sincerely,

Rojana Dhakal

Corresponding Author

**Editorial Corrections/ Changes and Justification**

| **SN** | **Feedback** | **Responses** |
| --- | --- | --- |
| 1. | Please ensure that your manuscript meets PLOS ONE's style requirements, including those for file naming. The PLOS ONE style templates can be found at | *A manuscript has been prepared based on the template provided in the given link.* |
| 2. | PLOS ONE does not copy edit accepted manuscripts (<https://journals.plos.org/plosone/s/criteria-for-publication#loc-5>). To that effect, please ensure that your submission is free of typos and grammatical errors. | *Typos and grammatical errors have been checked.* |
| 3. | We note that the grant information you provided in the ‘Funding Information’ and ‘Financial Disclosure’ sections do not match. When you resubmit, please ensure that you provide the correct grant numbers for the awards you received for your study in the ‘Funding Information’ section. | The financial disclosure statement has been updated and has been written in the cover letter. |
| 4. | Thank you for stating the following in the Acknowledgments Section of your manuscript:  “We would like to express our gratitude to the University Grants Commission (UGC-Nepal) of Nepal for awarding faculty research grants (FRG-73/74-HS-15) to carry out this research. In addition to this, we would like to express the deepest gratitude for study participants and B.Sc. Nursing 6th and 7th batch students of School of Health and Allied Sciences, Pokhara University for enormous support during the phase of data collection and intervention. RD, NN, PB, AG, NS received the grants to carry out the research from UGC-Nepal.” | The statement was revised and the funding information was deleted.  Any funding related information was removed from the manuscript. |
| 5. | Thank you for stating the following in your Competing Interests section. | Statement was modified as advised. |
| 6. | We note that you have indicated that data from this study are available upon request. PLOS only allows data to be available upon request if there are legal or ethical restrictions on sharing data publicly. For more information on unacceptable data access restrictions, please see | The data are available in the supporting information file. |
| 7. | Please include your tables as part of your main manuscript and remove the individual files. Please note that supplementary tables (should remain/ be uploaded) as separate ""supporting information"" files. | Tables are kept in the main section of the manuscript, and individual files are removed. |

**Addressing of Reviewer 1 comments**

| **SN** | **Feedback** | **Responses** |
| --- | --- | --- |
| 1 | Change title: The effect of educational intervention based on the theory of planned behavior ….: Stochastic controlled trial | *Thank you so much for the feedback. The title has been modified and now it seems: as in draft* |
| 2 | In the abstract, write the method of sampling. | *Please refer to line number 9-10. First, wards were randomly assigned in the intervention and control groups, then participants who satisfied inclusion criteria were purposefully chosen.* |
| 3 | Write the mean value and standard deviation for all model constructs. | *Since it looked hazy, we explained the outcomes in terms of significance.* |
| 4 | Explain more in the conclusion. | *Completely refined.* |
| 5 | Match the keywords with the mesh in PubMed | *Please refer lines 36-37. The key words changed and matched with the MeSH terms in PubMed.* |
| 6 | Explain the reason and importance of the study in the introduction. | *Added* |
| 7 | In the introduction, write a review of the texts and a review of other studies in this field. | *The review of other studies related to BSE or mammography screening and the intention to perform this behavior has been added in the introduction.* |
| 8 | Describe the application of the study in the introduction. | *Added* |
| 9 | Describe how to sample. | *Thank you for the feedback. The details of sample size calculation has been explained in lines 108-111.* |
| 10 | Write the entry and exit criteria in the working method. | *Thank you for the feedback. The inclusion and exclusion criteria of the study are explained in lines 114-118.* |
| 11 | Bring study restrictions | *We enrolled the participants based on their interests, and not randomized.* |
| 12 | Write how to determine the sample size | *We use OpenEPi for determining the sample size. This explain in lines 108-111.* |
| 13 | Explain tools, is it self-made or standardized? | *We adapted from literature review with iterative experts feedback.* |
| 14 | Write down the number of questions and the validity and reliability of the tool | *Thank you for the feedback. The number of questions has been added, validity and reliability of the tool please refer lines 130-134.* |
| 15 | Write how the tool scores. | *Thank you for the feedback. This had been added in the section of data analysis line 151-154. Refer table 1.* |
| 16 | Write down the intervention and the strategies used. | *Thank you for the feedback. Please refer lines 184-196.* |
| 17 | In the method, describe each learner for what type of analysis is used | *Independent t, and paired t wherever applicable* |
| 18 | In the results, write the standard deviation and mean values for all groups and variables | *Thank you for the feedback. As the tool is Likert Scale the analysis has been done considering the median score for descriptive analysis. For inferential analysis, independent and paired t-tests wherever applicable.* |
| 19 | Write down the strengths and weaknesses of the study. | *Added. Please refer 438-446* |
| 20 | Write study limitations | *Added. Please refer 438-446,* |
| 21 | Bring suggestions | *Given in conclusion* |
| 22 | In the discussion section, compare the results with other studies and write your reasons | *The literature very few on the related topic, so wherever possible reasons added. Please refer 417-426.* |

**Addressing of Reviewer 2 comments**

| **SN** | **Feedback** | **Responses** |
| --- | --- | --- |
| 1. | Introduction is too long. Please condense in to one half page. | *We thank the reviewer for pointing this comment. As reviewer first suggestion to add review or other studies related with topic, so some points are added and some data were deleted.* |
| 2. | Authors should be mention intervention studies based on TPB in introduction section. | *Thank you for the feedback. We added information of TPB in the line 94-95.* |
| 3. | Material and method a. Authors should be clarified setting samples were selected from a hospital, clinic or clinics: b. Authors should be clarified of how to educate illiterate people?:  c. Researchers need to figure out how to control confounders, especially in the control group: | a. *The samples were selected from a community.* b. *The illiterate people were educated with discussion, showing picture and demonstration of BSE in model. As in model can palpate the mass or lymphnodes or fibrosis in both breast.* c. *Potential confounding variable added in the test.* d. *Age matched with control and intervention.* e. *Two separate ward are chosen for intervention and control groups.* |
| 4 | Data collection instruments: Please mention numbers of items for constructs of TPB (attitude, subjective norms, perceived behavior control) and validity and reliability of its. | *Thank you for the feedback. The number of questions has been added, validity and reliability of the tool please refer lines 130-134.* |
| 5 | Discussion: Authors should be mention limitation of study. | *Limitation of the study has been added in the discussion section, please refer lines no 438-446.* |

**Addressing of Reviewer 3 comments**

| **SN** | **Feedback** | **Responses** |
| --- | --- | --- |
| 1. | Introduction  This test aids in the early detection of cases that are still curable. This refers to BSE or CBE or Mammogram. | *This refer to all three test.(CBE, BSE and mammography)* |
| 2 | Women should begin regular screening starting at age 45 years old. Women must have the chance to begin a yearly screening test between the ages of 40 and 44 years ole. Reference Please: | *This guidelines is provided by American Cancer Society. Please check reference number 7,8.* |
| 3 | It focuses on the women’s attitude and beliefs (21). This sentence is not clear. Please revise the sentence. | *The sentence has been revised.* |
| 4 | A study done in Kathmandu Nepal, found out of 500 women 3.4%, 7.2% and 14.4% of women had undergone mammography, clinical breast exam and breast self-examination respectively. I suggest write the abbreviation such as CBE: | *Changed as advised.* |
| 5 | If a women believes she is at risk of breast cancer, she is more likely to undertake screening measures. Reference please. | *Please find the reference number 15 study done in Kathmandu Nepal to identify the factors associated with breast cancer screening intention.* |
| 6 | To date, there has been very little study on the effectiveness of educational intervention in determining women’s desire to go for breast cancer screening in Nepal or world: | ***This is the own sentence. It related with Nepal*** |
| 7 | ***Study design and setting***  A quasi-experimental controlled time series study with a control group design was conducted to assess the effectiveness of educational intervention on the intent to perform breast cancer screening among women in Pokhara, Nepal. Please remove “with a control group”. It was repeated | *Repeated information was deleted as advised.* |
| 8 | Respondents were selected by using purposive sampling. The sample size was determined using the Open Epi sample size calculator for a cross sectional study with a power of 80%, and a confidence level of 95%, with an assumption of study from mammography utilization (25). Why “ for a cross sectional study”?? | *Thank you for the feedback. Open Epi Sample size calculator is used for cross sectional, cohort and Randomized clinical trials.* |
| 9 | ***Data Collection instruments***  The calculated sample size was 154 in the exposed group and 154 in the non-exposed group. I suggest using consistent terminology throughout manuscript: interventional, exposed/intervention and non-exposed/control:  The questionnaire was developed by the researchers using the theory of planned behavior principles, considerable reviews, and expert comments. I suggest using constructs instead of principles: | *Thank you for the feedback. We used consistent same terminology intervention and control.*  *Thank you for the feedback. We changed terminology principles into constructs.* |
| 10 | **Outcome Variables**  The intention to go for monthly breast self-examination and mammography every two years was the primary result of this research. There is secondary outcome as well?: | *The secondary outcome was practicing screening behavior; regular BSE and mammography. Which is not included in this article.* |
| 11 | The theory of planned construction of behavior intention based on score cut-off points. It is not clear: | *The sentence was removed from there and score-related information was added to the exposure variables sections.* |
| 12 | **Data Collection**  Every time the same tool was used to collect the information, the intervention group was given a repeated intervention in a time frame. Please clarify how much time before using the tool occurred intervention: | *Sentence was modified. The data was collected for four times from same individuals, baseline, at 4 months, 8 months and 12 months. The intervention was provided for three times after collecting baseline data, at 4 months and 8 months.* |
| 13 | **Ethics Statement**  The comparison group provided the same informative session at the end of the research. The comparison group was provided the same informative session at the end of the research.  At the conclusion of the research, the control group offered the identical instructional session. The comparison group was provided the same informative session at the end of the research. I think two sentences offer the same meaning. Please revise it: | *The repeated information was deleted.*  *Thank you for the feedback. The sentence was corrected and repeated information was deleted.* |
| 14 | **Data analysis**  Tables were used to represent the information. Please remove it. | *Removed the sentence as advised.* |
| 15. | **Results**  I suggest removing the first sentence.  The average age in the interventional and control group was49. I suggest writing about baseline features not the intervention and control groups.  From 35.6 percent at baseline 35.6%:  High intention was found in subsequent posttest 1 (87.2%) and dropped in Posttest 2 (37.2%) and again increased in posttest 3 (83.6%) in which group?  Further, results showed that after intervention at 4 months and 12 months, significant differences were found between the intervention and control except in the except in the 8 months regards referents with peer. It is not clear.  Then, there was no significant change observed even after the intervention was given in the control factors in the experimental groups. Please revise it.  Generally, I suggest removing repeated sentences and reduce the results section. | *Removed the first sentence as advised.*  *Thank you for the information. The study compared the intervention and control groups. The comparison has been shown to show that there were no differences among the demographic variables.*  *Changed as Advised.*  *Sentence modified. .*    *Sentence modified.*  *Sentence modified.*  *Repeated information was carefully removed and reduce the results section.* |
| 16. | **Discussion**  I suggest, in the first paragraph, report the main results of the study. And the explanation about TPB move to method or remove here:  I suggest removing repeated sentences and directly discussing the main points of the issue in the women. The discussion needs to be improve and reduce. | *Changed as advised*  *Thank you for the feedback. We removed the repeated sentence and discuss the main issues of the study.* |
