# Supplementary material for: Attitude sustains longer than subjective norm and perceived behavioral control: Results of breast cancer screening educational intervention
Source: PLoS One. 2023 Feb 10;18(2):e0281184. doi: 10.1371/journal.pone.0281184 (PMC9916560; doi:10.1371/journal.pone.0281184)
Supplement: Attachment — Submitted filename: Response to Reviewers.docx [file pone.0281184.s004.docx]

To:

The Academic Editor

Plos One

From:

Rojana Dhakal

6^th^ December, 2022

Dear Sir/Madam,

We would like to thank the reviewers for their generous comments on the manuscript entitled “***Attitude sustains longer than subjective norm and perceived behavioral control: Results of Breast Cancer Screening Educational Intervention”.*** *W*e have revised the manuscript, including the title. We address all the comments raised by both the editor and the reviewers. In addition, one of the authors (Niranjan Koirala), affiliated institution address has changed because he recently joined to another institution.

Sincerely,

Rojana Dhakal

Corresponding Author

**Editorial Corrections/ Changes and Justification**

| **SN** | **Feedback** | **Responses** |
| --- | --- | --- |
| 1. | **Journal Requirements**  Please review your reference list to ensure that it is complete and correct. If you have cited papers that have been retracted, please include the rationale for doing so in the manuscript text, or remove these references and replace them with relevant current references. Any changes to the reference list should be mentioned in the rebuttal letter that accompanies your revised manuscript. If you need to cite a retracted article, indicate the article’s retracted status in the References list and also include a citation and full reference for the retraction notice. | *Reference list has been checked for completeness and revised it. Probably, there isn’t any retracted paper cited in paper.* |
| 2. | Ensure that the revised manuscript is edited for LANGUAGE. | *The revised manuscript has been edited for language. At first, two authors (RD, CA) edited the manuscript in detail and rigorously. Then our another three authors (PB,NK,NS) checked for grammar and syntax in Quilbot (online software), and then again checked by three authors (RD,CA,NS) for grammar, language/syntax and the meaning of the statistical findings. RD and CA again checked before final submission.* |

**Addressing of Reviewer 1 comments**

| **SN** | **Feedback** | **Responses** |
| --- | --- | --- |
| 1 | "Change title: The effect of educational intervention based on the theory of planned behavior ....: Stochastic controlled trial." | *Thank you so much for the feedback. The numbers of possible title has been discussed among the authors. The title has been changes, with the concession among all the authors, and it now seems: “as in draft”. We revised it to make compatible with our main conclusion, and the variables (the intent covers our three variables of TPB), and hope is also catchy.* |
| 2 | "Theory of planned behavior" is not a MESH term." | *The Phrase has been removed.* |
| 3 | "Separate sub-heading for inclusion and exclusion criteria." | *Changes have been made accordingly. Please refer lines no 148-155.* |
| 4 | "Indicate clearly all the relevant p-values in the description of respective tables." | *Changes have been made accordingly in the descriptions of the respective tables and in text, including in abstract.* |

**Addressing of Reviewer 3 comments**

| **SN** | **Feedback** | **Responses** |
| --- | --- | --- |
| 1. | "Overall English editing is needed?" e.g. "Introduction: "These" not "This"." | *The revised manuscript has been edited for language. At first, two authors (RD, CA) edited the manuscript in detail and rigorously. Then our another three authors (PB,NK,NS) checked for grammar and syntax in Quilbot (online software), and then again checked by three authors (RD,CA,NS) for grammar, language/syntax and the meaning of the statistical findings. To note the errors, hard copies have been checked. RD and CA rechecked before the final submission.* |
